# Integrated whole genome microarray analysis and immunohistochemical assay identifies COL11A1, GJB2 and CTRL as predictive biomarkers for pancreatic cancer

**DOI:** 10.1186/s12935-018-0669-x

**Published:** 2018-11-06

**Authors:** Defeng Sun, Haoyi Jin, Jun Zhang, Xiaodong Tan

**Affiliations:** 1 Shengjing Hospital of China Medical University, Shenyang, 110004 People’s Republic of China; 2 Gastric Cancer Department, Liaoning Province Cancer Hospital & Institute (Cancer Hospital of China Medical University), Shenyang, 110004 People’s Republic of China; 3 Thyroid and Pancreatic Surgery Ward, Shengjing Hospital of China Medical University, Shenyang, 110004 People’s Republic of China

**Keywords:** Bioinformatics, Pancreatic cancer, Biomarker, COL11A1, GJB2, CTRL

## Abstract

**Background:**

Pancreatic cancer is characterized by its unsatisfying early detection rate, rapid disease progression and poor prognosis. Further studies on molecular mechanism and novel predictive biomarkers for pancreatic cancer based on a large sample volume are required.

**Methods:**

Multiple bioinformatic analysis tools were utilized for identification and characterization of differentially expressed genes (DEGs) from a merged microarray data (100 pancreatic cancer samples and 62 normal samples). Data from the GEO and TCGA database was utilized to validate the diagnostic and prognostic value of the top 5 upregulated/downregulated DEGs. Immunohistochemical assay (46 paired pancreatic and para- cancerous samples) was utilized to validate the expression and prognostic value of COL11A1, GJB2 and CTRL from the identified DEGs.

**Results:**

A total number of 300 DEGs were identified from the merged microarray data of 100 pancreatic cancer samples and 62 normal samples. These DEGs were closely correlated with the biological characteristics of pancreatic cancer. The top 5 upregulated/downregulated DEGs showed good individual diagnostic/prognostic value and better combined diagnostic/prognostic value. Validation of COL11A1, GJB2 and CTRL with immunohistochemical assay showed consistent expression level with bioinformatics analysis and promising prognostic value.

**Conclusions:**

Merged microarray data with bigger sample volume could reflect the biological characteristics of pancreatic cancer more effectively and accurately. COL11A1, GJB2 and CTRL are novel predictive biomarkers for pancreatic cancer.

**Electronic supplementary material:**

The online version of this article (10.1186/s12935-018-0669-x) contains supplementary material, which is available to authorized users.

## Background

Pancreatic cancer is characterized by its unsatisfying early detection rate, rapid disease progression and poor prognosis [1, 2]. Multiple aberrantly expressed genes and dysregulated signaling pathways have been reported to play critical roles in the development of pancreatic cancer [3–7]. However, the underlying molecular mechanism of pancreatic cancer is still not fully understood. Whole genome microarray is an effective way to analyze the expression profile of a human being in cell or tissue level [8]. Multiple studies and government supported projects like The Cancer Genome Atlas (TCGA) have performed whole genome microarray of patients’ pancreatic cancer tissues and its paired adjacent normal pancreatic tissues to identify the differentially expressed genes (DEGs) [9–11]. These DEGs could be potential key regulators in disease progression and predictive biomarkers for pancreatic cancer [12–14]. However, the accuracy and efficacy of aforementioned studies are compromised by their limited sample volume. The results obtained from studies with limited sample volume might be unrepresentative and biased. Comparing with other cancers like lung cancer or breast cancer, pancreatic cancer is a rather rare cancer type. Therefore, an integrated analysis of the exisiting whole genome microarray data is a more practical and cost effective way to overcome the aforementioned shortcomings [15–17].

In this study, we selected and further merged the data of 162 samples from 3 qualified Gene Expression Omnibus (GEO) datasets (GSE15471, GSE16515 and GSE32676) for integral analysis. Gene Set Enrichment Analysis (GSEA) was performed to evaluate the efficacy and accuracy of our merged data to reflect the biological differences between cancer and normal tissues [18]. A total number of 300 DEGs were identified from the merged data (|log_2_^Fold change^| > 1.5 and adjusted p < 0.05). Gene Ontology (GO) analysis and Kyoto Encyclopedia of Genes and Genomes (KEGG) pathway analysis were performed to analyze the functional classification and enriched signaling pathways of the DEGs [19–22]. Protein–protein interaction (PPI) analysis was performed to visualize the interaction network of identified DEGs. Individual and combined diagnostic value of the top 5 upregulated and downregulated genes were evaluated with receiver operating characteristic curve (ROC) analysis. We further validated the expression level and performed survival analysis of these 10 genes with the data from TCGA database. Finally, we selected and validated the clinical predictive value of COL11A1 (collagen alpha-1(XI) chain), GJB2 (gap junction beta-2 protein) and CTRL (chymotrypsin-like protease CTRL-1) with immunohistochemical (IHC) analysis of 46 paired pancreatic cancer and para-cancerous tissue sections.

## Methods

### GEO datasets selection

The following standards were applied for qualified GEO datasets selection. (1) Whole genome microarrays of pancreatic cancer tissues and paired adjacent normal pancreatic tissues were included only. Microarrays containing samples as pancreatic cancer cell lines or pancreatic organoids were excluded. (2) Whole genome microarrays with sample volume > 30 samples were included only. Datasets with sample volume < 30 samples were excluded to avoid the inclusion of unrepresentative data. (3) Published whole genome microarrays were included only for better quality control and repeatability. (4) Whole genome microarrays of the same platform were included only (GPL570[HG-U133_Plus_2]Affymetrix Human Genome U133 Plus 2.0 Array). Datasets from other platforms were excluded to avoid the potential bias caused by technological difference between different platforms. In accordance with these standards, three qualified datasets (GSE15471, GSE16515 and GSE32676) were selected for further analysis.

### Data normalization and merging

Raw data of the selected microarrays were extracted with the R language package affy. Extracted expression data were normalized and transformed to log2 based logarithm with the rma function of affy package. Batch effect was excluded with the R language package sva before the aforementioned data were merged into one dataset.

### Gene Set Enrichment Analysis

Efficacy and accuracy of the merged data to reflect the biological differences between tumor and normal groups were evaluated with Gene Set Enrichment Analysis (GSEA, Broad Institute, http://www.broadinstitute.org/gsea/index.jsp) in accordance with the official tutorial.

### Identification of differentially expressed genes

The R language package limma was utilized for the calculation of DEGs (Fold change = Pancreatic cancer sample expression/paired adjacent normal pancreatic tissue sample expression, |log_2_^fold change^| > 1.5 and adjusted p < 0.05). For repeated gene expression data, the one with smaller p value was used for the downstream analysis. The expression level of DEGs was visualized with R language package ggplot2, pheatmap and GraphPad Prism 7 software.

### GO analysis and KEGG analysis

The R language package clusterProfiler and online database Database for Annotation, Visualization and Integrated Discovery (DAVID, https://david.ncifcrf.gov/) was utilized for GO analysis and KEGG analysis of the DEGs. The results were visualized with R language.

### Protein–protein interaction network analysis

The PPI network of identified DEGs was calculated with STRING database (https://string-db.org/cgi/input.pl) and visualized with Cytoscape software in accordance with the official tutorial [23].

### ROC analysis of selected differentially expressed genes

Diagnostic value of the top 5 upregulated and downregulated genes from the identified DEGs was calculated with ROC analysis. ROC analysis of individual genes and combined genes were performed with SPSS 19.0 and visualized with GraphPad Prism 7. Diagnostic model of combined genes was established by binary logistic regression with SPSS 19.0.

### Validation with TCGA database and survival analysis

The expression level of the top 5 upregulated and downregulated genes from the identified DEGs was validated with microarray data from TCGA database and further visualized with GraphPad Prism 7. Prognostic value of these 10 selected genes was evaluated with survival analysis. Survival analysis was performed with OncoLnc platform (http://www.oncolnc.org/) utilizing clinical data from TCGA database.

### Immunohistochemical assay

IHC analysis of the patients’ pancreatic cancer tissue sections and its paired para-cancerous tissue sections was performed as previously described [24]. Antibody against COL11A1, GJB2 and CTRL was used at the dilution range of 1:300 (Abcam, Cambridge, UK). The stained sections were evaluated by two different specialists. The postoperative survival rate of patients with different expression level of these two genes was visualized with GraphPad Prism 7.

### Statistical analysis

Student’s t-test was performed with IBM SPSS Statistics version 19.0. p < 0.05 was considered statistically significant.

## Results

### Identification of differentially expressed genes from merged microarray dataset

**Fig. 1:**
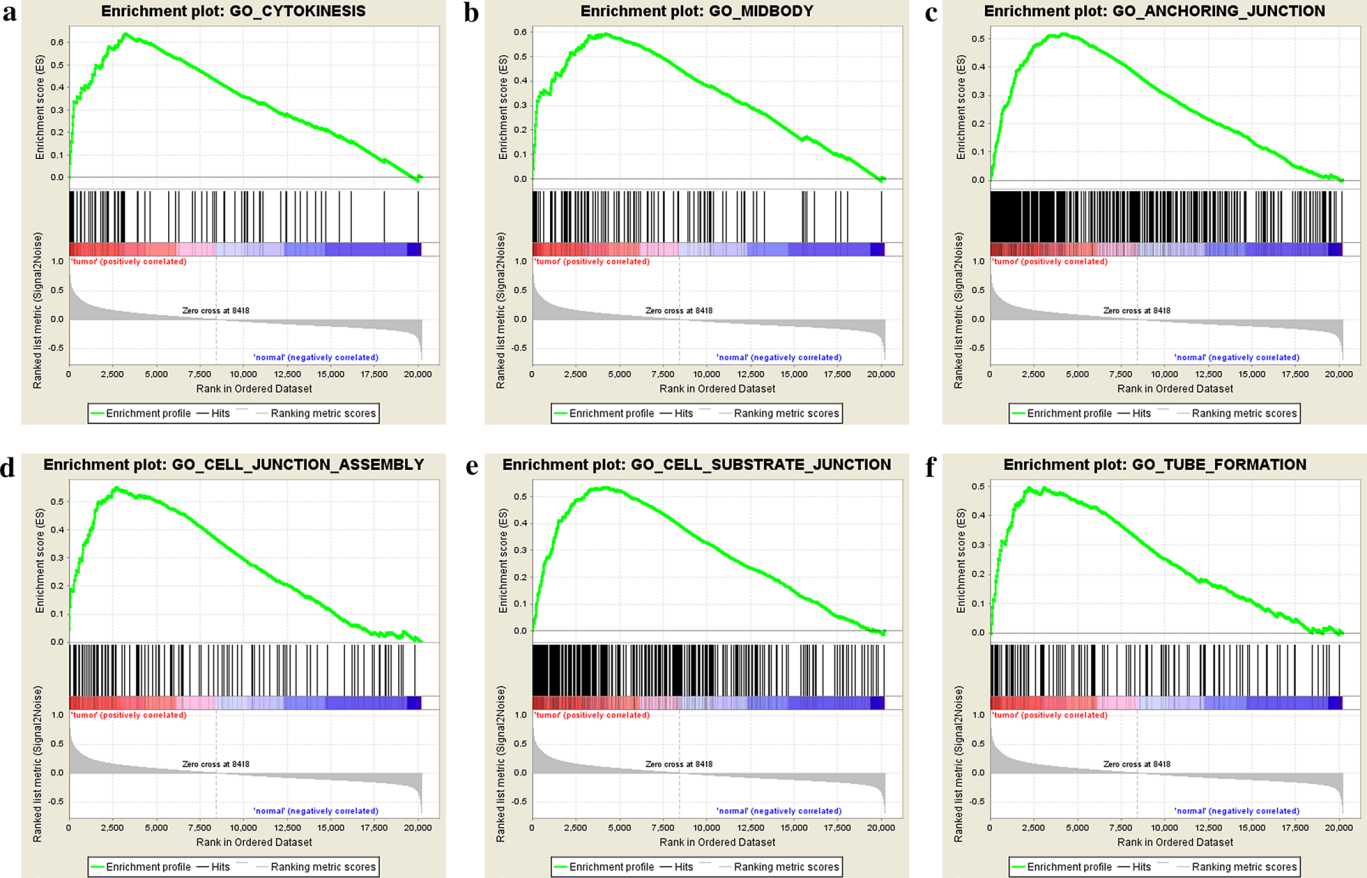
Identification of enriched genesets in cancer group with GSEA analysis of the merged microarray **a** cytokinesis, **b** midbody, **c** anchoring junction, **d** cell junction assembly, **e** cell substrate junction, **f** tube formation. *GSEA* Gene Set Enrichment Analysis

**Fig. 2:**
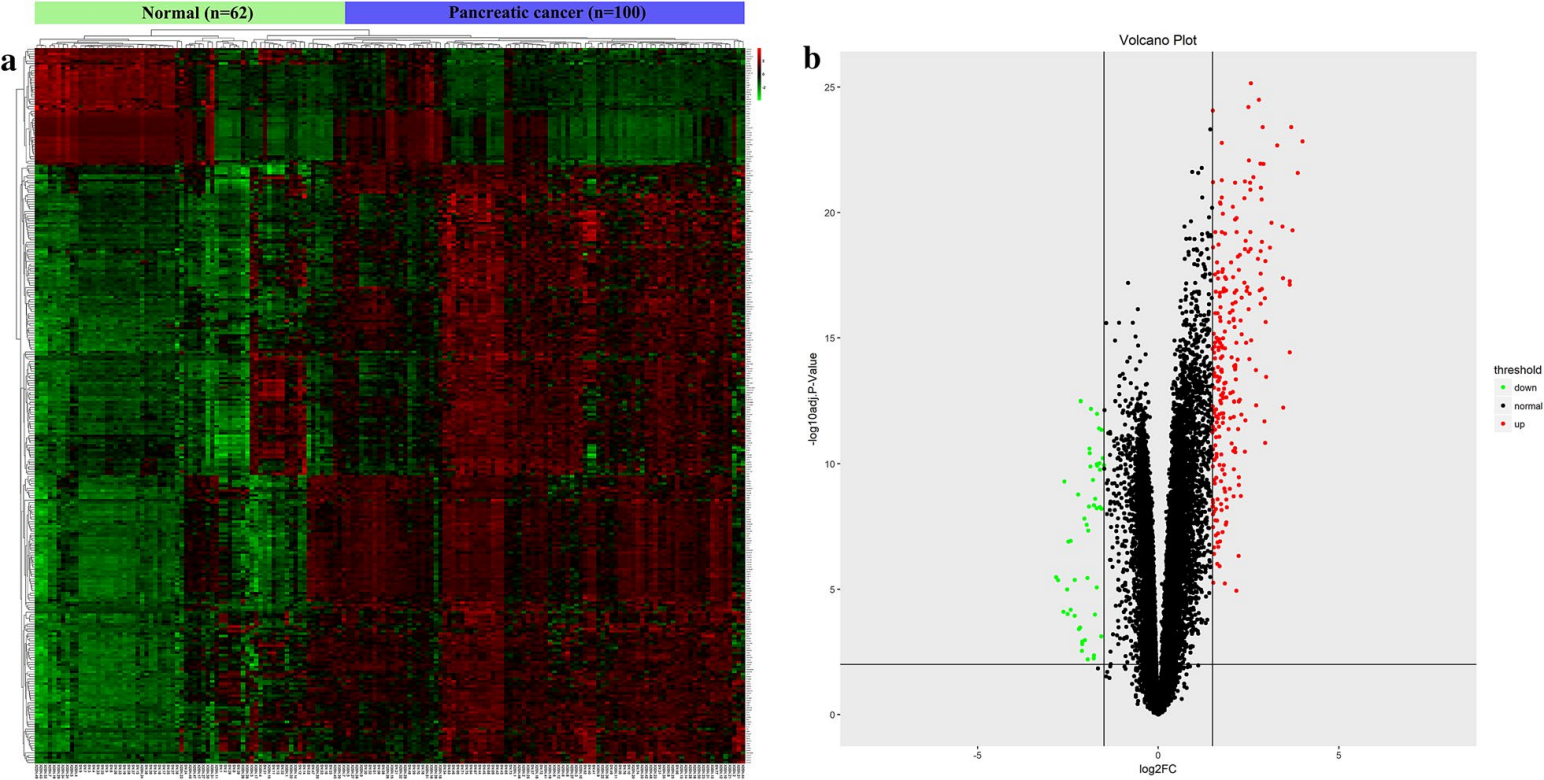
Heatmap and volcano map of the identified DEGs **a** Heatmap of the DEGs, **b** volcano map of the DEGs. Red, green and black color represents relatively high, low and equal expression of genes in the corresponding group. *DEGs* differentially expressed genes

Three qualified GEO datasets were merged into one dataset which contains microarray data of 100 pancreatic cancer tissue samples and 62 normal pancreas tissue samples. GSEA analysis of the merged dataset showed that the tumor group was enriched in genes regulating cell division (cytokinesis, midbody), cell junction (anchoring junction, cell junction assembly, cell substrate junction) and tube formation (Fig. 1a–f). These enriched gene sets were closely correlated with cell proliferation, migration, invasion and angiogenesis. The aforementioned results suggested that our merged data was qualified to reflect the biological characteristics and expression profile of cancer samples. A total number of 300 DEGs was further identified from the merged microarray data, of which 251 were upregulated and 49 were downregulated in pancreatic cancer samples (Additional file 1). Identified DEGs were visualized with heatmap and volcano map (Fig. 2a and b).

### GO analysis and KEGG analysis of the identified differentially expressed genes

**Fig. 3:**
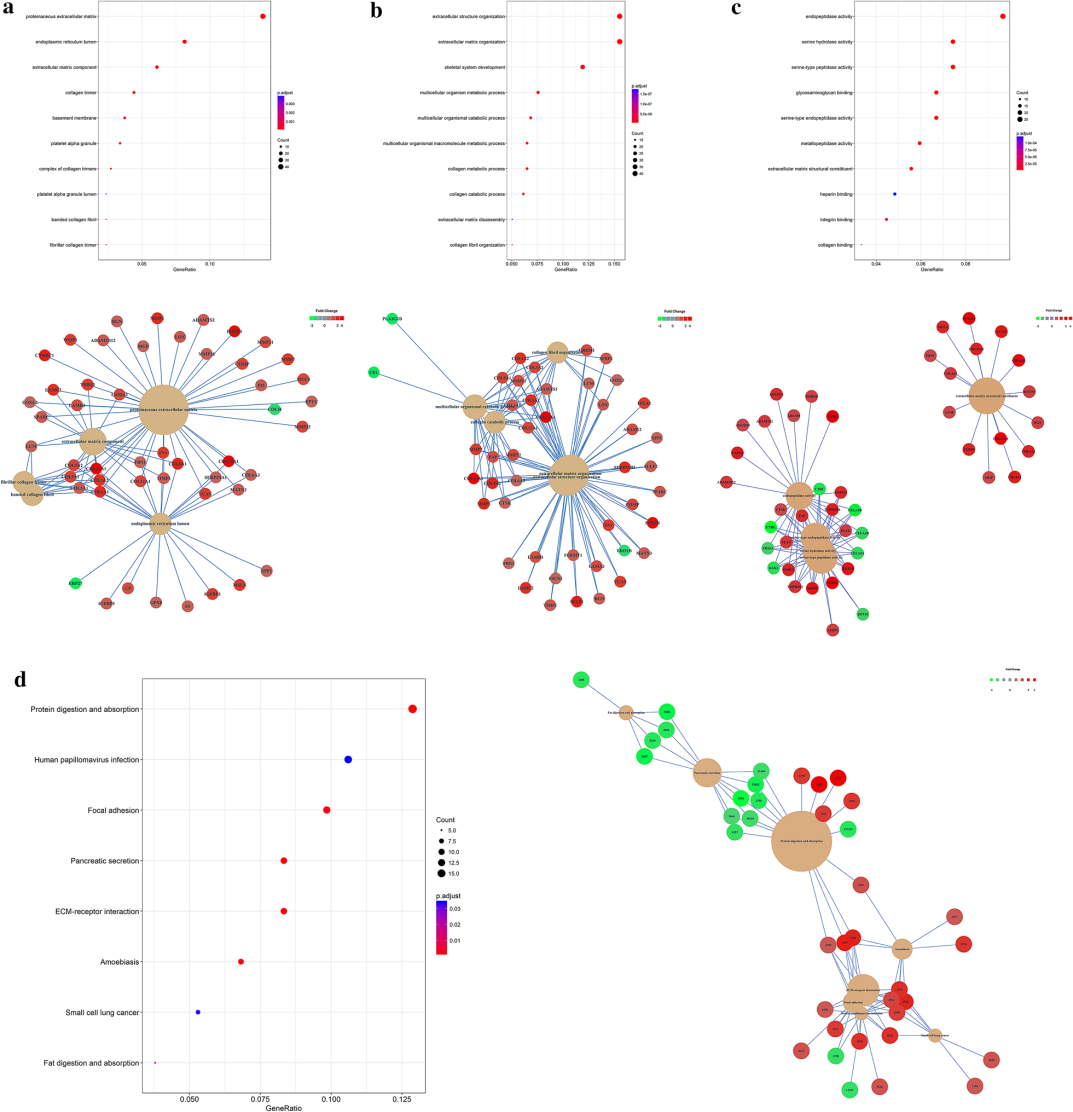
GO analysis and KEGG pathway analysis of the identified DEGs **a** GO analysis of the DEGs in cellular component, **b** GO analysis of the DEGs in biological process, **c** GO analysis of the DEGs in molecular function, **d** KEGG pathway analysis of the DEGs. *GO* Gene Ontology, *KEGG* Kyoto Encyclopedia of Genes and Genomes, *DEGs* differentially expressed genes

GO analysis and KEGG analysis were performed to analyze the functional classification and signaling pathway enrichment of the identified DEGs. The results showed that the DEGs were closely correlated with extracellular environment reorganization in cellular component (CC) and biological process (BP) classification (Fig. 3a and b). For molecular function (MF) classification, the DEGs were enriched in multiple peptidase activity and integrin binding (Fig. 3c). These results suggested that the DEGs were closely correlated with extracellular matrix (ECM) degradation and remodeling which is the essential step for local invasion and distant metastasis. The results from KEGG analysis showed that the DEGs were significantly enriched in signaling pathways of pancreatic secretion, fat/protein digestion and absorption (Fig. 3d). This suggested that the development of pancreatic cancer is characterized by the loss of pancreas’s normal physiological functions. In accordance with GO analysis, the DEGs were closely correlated with signaling pathways regulating ECM-receptor interaction and focal adhesion.

### Protein–protein interaction analysis of the identified differentially expressed genes

**Fig. 4:**
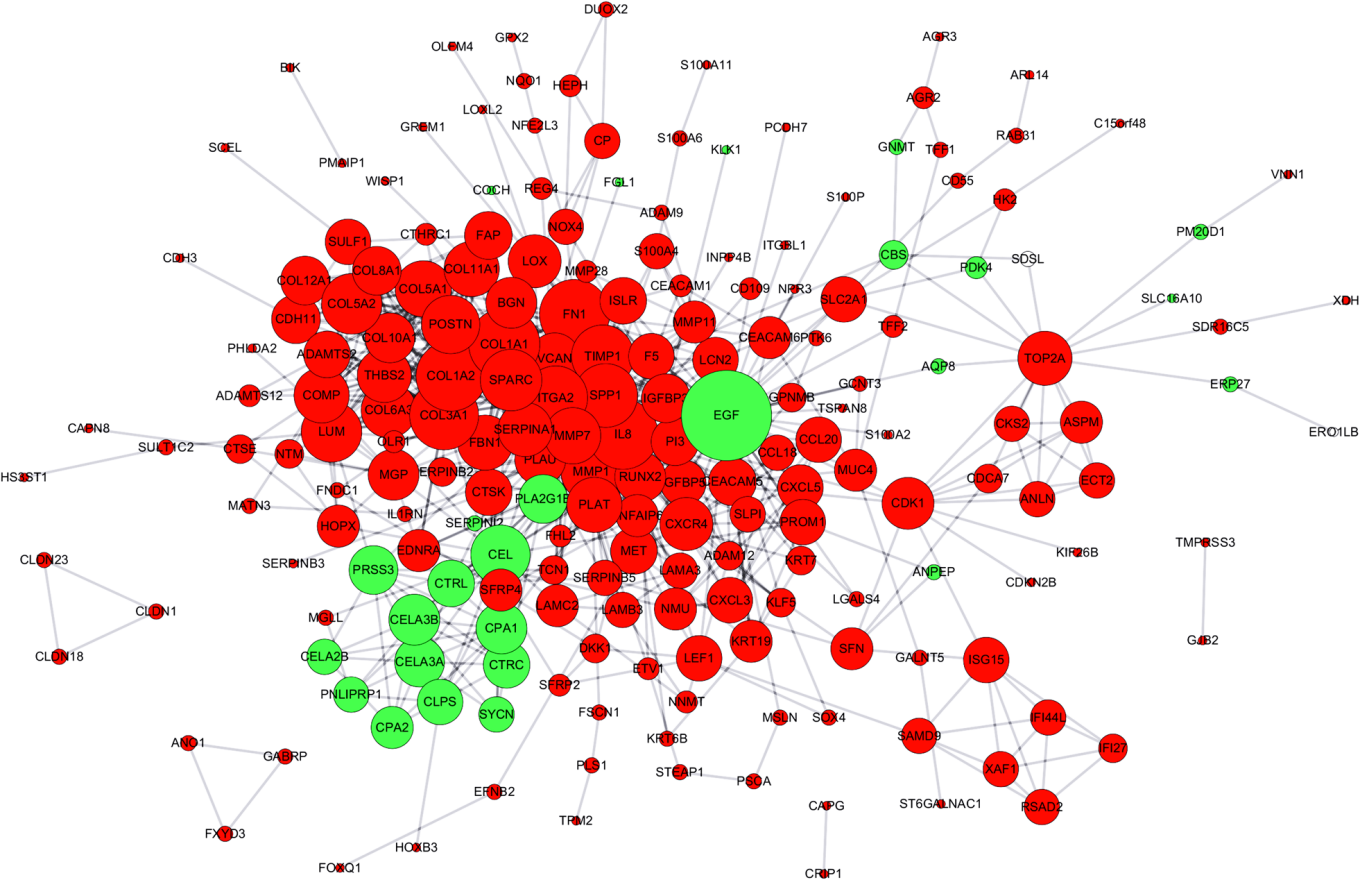
PPI network analysis of the identified DEGs. Red and green color represents upregulated and downregulated DEGs in tumor group. The size of the node is determined by the number of its interaction with other nodes. *PPI* protein–protein interaction, *DEGs* differentially expressed genes

We utilized STRING database to analyze the PPI network of the DEGs to identify the key genes and their interactions in pancreatic cancer progression. The visualized results showed that epidermal growth factor (EGF) was located in the core of our PPI network (Fig. 4). COL11A1, COL10A1 from the top 5 upregulated genes and CTRL, SYCN, PNLIPRP1 from the top 5 downregulated genes were also found to be the key genes in the PPI network (Table 1).

**Table 1:** Top 5 upregulated and downregulated genes of the identified differentially expressed genes

Top 5 upregulated			Top 5 downregulated		
Gene symbol	Log_2_^Fold change^	Adjusted p value	Gene symbol	Log_2_^Fold change^	Adjusted p value
S100P	3.99423324	1.47E−23	SERPINI2	− 2.82817983	3.33E−06
COL10A1	3.860974743	2.67E−22	CTRL	− 2.77212775	4.45E−06
COL11A1	3.71532016	5.14E−20	SYCN	− 2.6228281	7.92E−05
KRT19	3.679278432	4.01E−24	SPX	− 2.594566083	5.17E−10
CEACAM5	3.639738913	3.80E−15	PNLIPRP1	− 2.520394006	1.02E−05

### Potential clinical value of the top 5 upregulated and downregulated differentially expressed genes

**Fig. 5:**
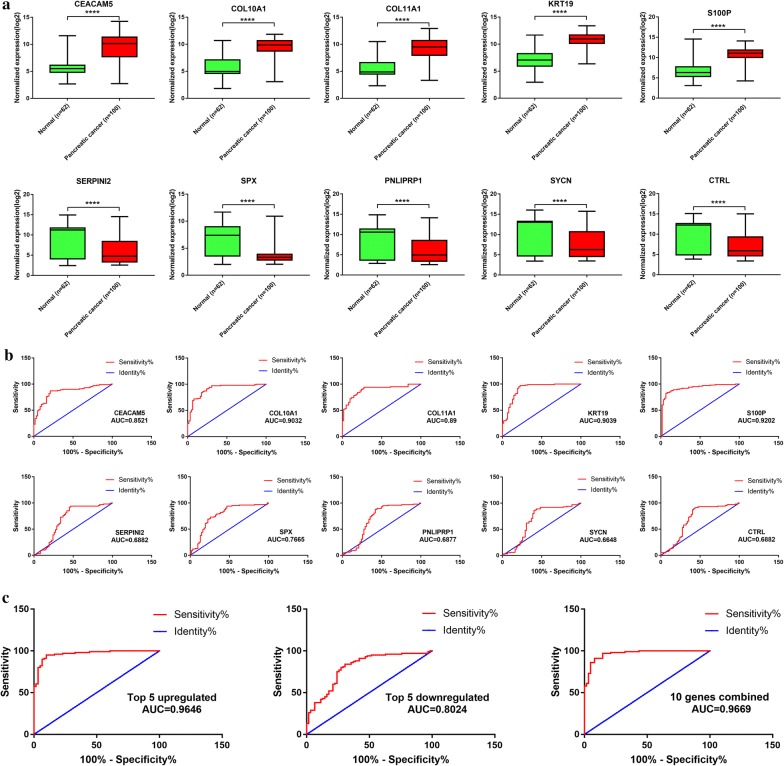
Expression level and diagnostic value of the top 5 upregulated and downregulated DEGs in merged data **a** expression level of the selected DEGs in merged data. Top 5 upregulated genes: CEACAM5, KRT19, COL11A1, COL10A1 and S100P. Top 5 downregulated genes: SERPINI2, CTRL, SYCN, SPX and PNLIPRP1, **b** ROC analysis of the selected DEGs individually, **c** ROC analysis of the selected DEGs combined. *AUC* area under curve, *DEGs* differentially expressed genes

**Fig. 6:**
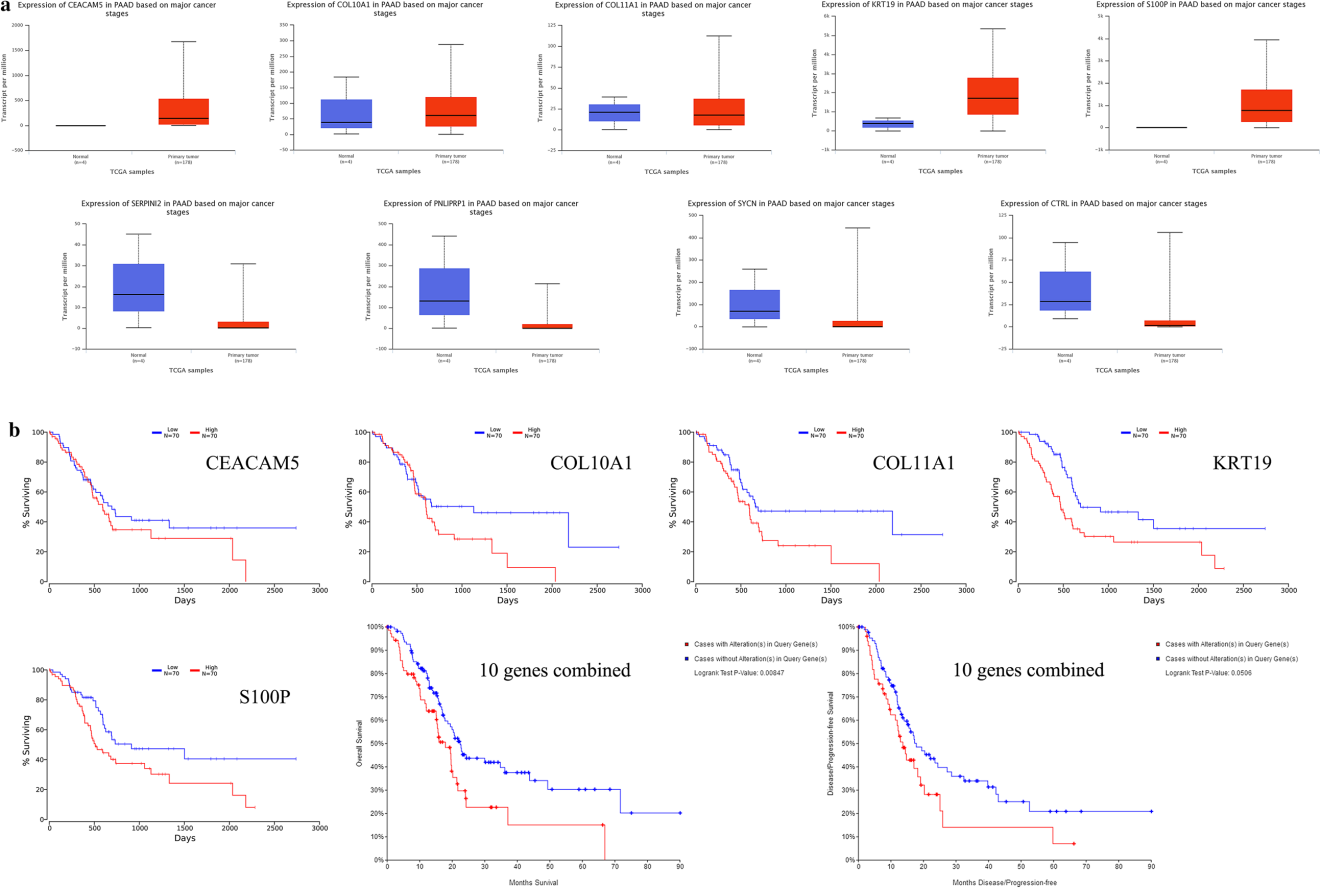
Expression level and prognostic value of the top 5 upregulated and downregulated DEGs in TCGA database **a** expression level of the selected DEGs in TCGA. Expression data of SPX is absent, **b** survival analysis of the top 5 upregulated DEGs individually, **c** survival and disease free analysis of the selected DEGs combined. *DEGs* differentially expressed genes

The top 5 upregulated and downregulated DEGs were selected for further validation of their potential diagnostic value (Fig. 5a and b). The results of ROC analysis indicated that these 5 upregulated DEGs possessed higher individual diagnostic value than those 5 downregulated DEGs. We established a diagnostic panel of these genes combined with binary logistic regression. These 10 genes showed better combined diagnostic value than individually (Fig. 5c). We further validated the expression level of these 10 genes with the data from TCGA database. The results showed similar expression level of these selected DEGs except no expression data of SPX was found (Fig. 6a). Survival analysis of the top 5 upregulated DEGs showed promising prognostic value (Fig. 6b). Combined analysis of these 10 genes showed that dysregulation of these 10 genes was closely correlated with poorer overall survival rate and disease free survival rate (Fig. 6c).

### Validation of COL11A1, GJB2 and CTRL with immunohistochemical assay of clinical samples

**Fig. 7:**
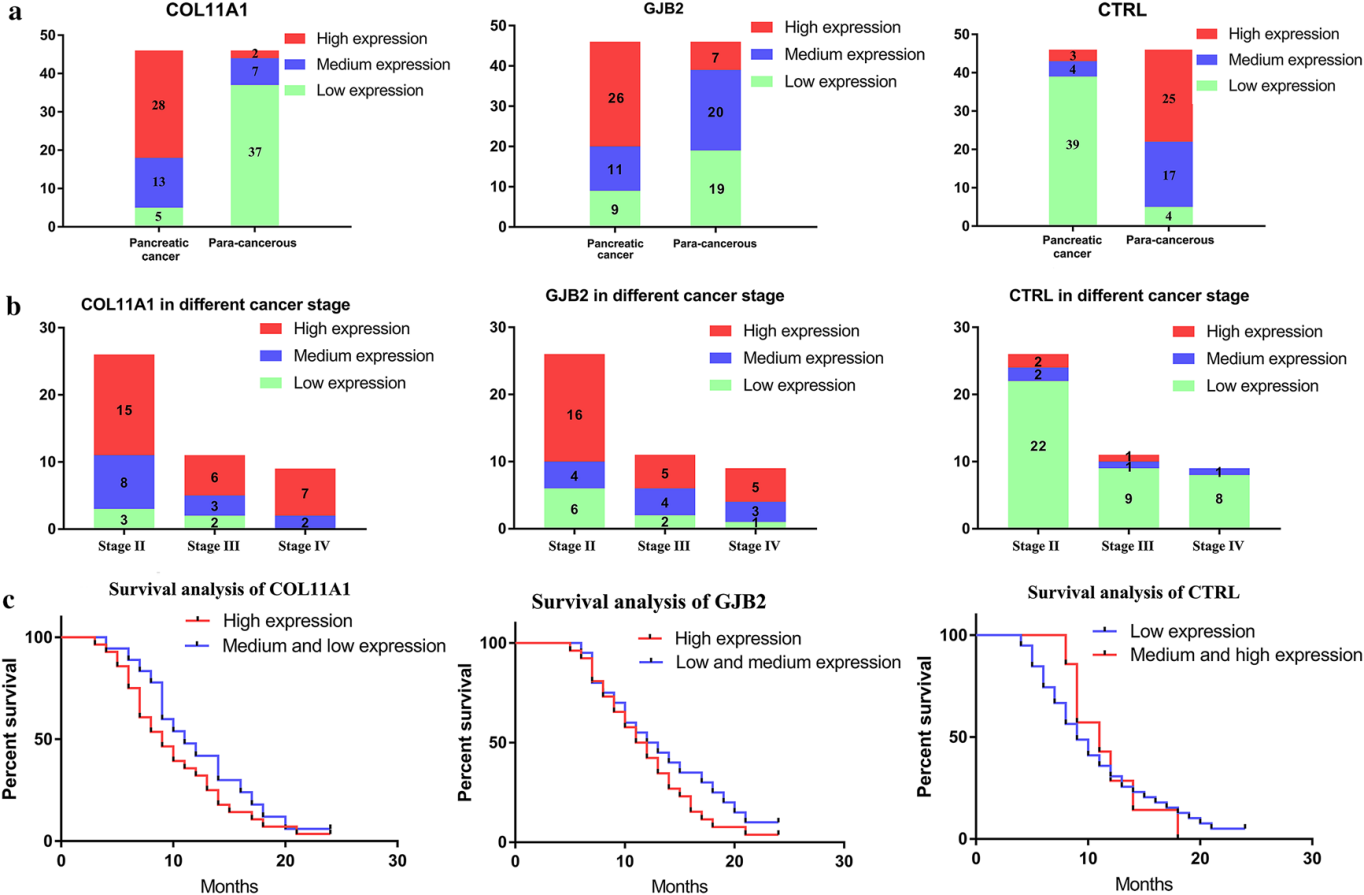
Validation of COL11A1, GJB2 and CTRL with immunohistochemical assay of clinical samples **a** expression level of COL11A1, GJB and CTRL in 46 paired pancreatic cancer and para-cancerous tissue sections, **b** expression level of COL11A1 GJB2 and CTRL in different cancer stages, **c** survival analysis of patients with different expression level of COL11A1, GJB2 and CTRL

We validated the expression level of COL11A1, GJB2 and CTRL with IHC assay of 46 paired pancreatic cancer and para-cancerous tissue sections (Table 2). COL11A1 and CTRL were chosen for further validation from the top 5 upregulated and downregulated DEGs as they were also identified as key genes in our PPI analysis and had suitable antibodies for IHC assay. GJB2 was chosen as dysregulation of gap junction proteins is closely correlated with cancer progression and there was no published papers regarding the role of GJB2 in pancreatic cancer. IHC analysis showed consistent results with our bioinformatic analysis (Fig. 7a). Representative images of the IHC assay were uploaded in supplementary files (Additional file 2). We also analyzed the expression level of these three genes in different cancer stages (Fig. 7b). Next, we investigated the prognostic value of COL11A1, GJB2 and CTRL with our clinical and follow-up data (Fig. 7c). The result showed that high expression level of COL11A1, GJB2 or low expression level of CTRL in pancreatic cancer tissue sections indicated poorer prognosis and less survival rate. These results suggested promising clinical predictive value of these three genes.

**Table 2:** Clinical characteristics of 46 paired pancreatic cancer tissue sections and para-cancerous tissue sections

Patients characteristics	Number	Percentage
Gender		
Male	25	54%
Female	21	46%
Age		
Mean (years)	58.36 ± 9.48	
Histopathologic subtype		
Well-differentiated adenocarcinomas	19	41%
Moderately differentiated adenocarcinomas	10	22%
Poorly differentiated adenocarcinomas	17	37%
TNM staging		
T2N0M0	11	24%
T2N1M0	3	7%
T2N1M1	1	2%
T3N0M0	14	30%
T3N1M0	7	15%
T3N0M1	5	11%
T3N1M1	3	7%
T4N0M0	1	2%
T4N1M0	1	2%
Local invasion and distant metastasis		
Local invasion	14	31%
Regional lymph node metastasis	15	33%
Liver metastasis	6	13%
Ascites metastasis	2	4%

## Discussion

In the present study, we utilized multiple bioinformatic analysis and IHC analysis to investigate two key hypotheses. (1) Merged microarray data with bigger sample volume could reflect the biological characteristics of cancer group and normal group more effectively and accurately. We could obtain a more representative and accurate results from the merged microarray data. (2) DEGs identified from the merged microarray data hold promising potential as key regulators and predictive biomarkers for pancreatic cancer. GSEA analysis of the merged data showed that the cancer group was enriched in genes closely correlated with cell proliferation, migration, invasion and angiogenesis. These results suggested that our merged data was qualified to reflect the characteristic expression profile of cancer and normal group. GO analysis and KEGG pathway analysis of the identified DEGs showed that they were closely correlated with ECM regulation and pancreatic secretion. These results were consistent with the clinical features of pancreatic cancer which is characterized by early local invasion/distant metastasis and loss of normal exocrine function. PPI network analysis showed that EGF was the core in the interaction network of identified DEGs. Multiple clinical trials on investigating the efficacy of combining Cetuximab (a targeted antibody against EGFR) with currently applied therapies have been reported [25–27]. All of the aforementioned results suggested that our merged microarray data could effectively and accurately reflect the characteristic biological differences between pancreatic cancer tissues and adjacent normal pancreatic tissues. These identified DEGs of our study could be potential candidates for novel predictive biomarkers or targets for chemotherapy. Therefore, we first validated the diagnostic and prognostic value of the top 5 upregulated and down regulated DEGs with public data from the GEO and TCGA database. We further validated the clinical predictive value of COL11A1, GJB2 and CTRL with IHC analysis of our own clinical derived sections. COL11A1 has been reported to be secreted by cancer associated fibroblasts and is closely correlated with the progression of multiple cancer types. The relationship between GJB2, CTRL and cancer progression was not reported. Furthermore, no published paper evaluated the expression and predictive value of COL11A1, GJB2 and CTRL in pancreatic cancer. Here, the results of IHC assay and survival analysis showed that these three genes could serve as predictive biomarkers for pancreatic cancer. Further functional analysis these three genes with in vitro and in vivo experiments are required in our future studies.

## Conclusions

In this study, we showed that merged microarray data with bigger sample volume could reflect the biological characteristics of pancreatic cancer more effectively and accurately. Results of bioinformatic analysis and IHC analysis suggested that COL11A1, GJB2 and CTRL are novel predictive biomarkers for pancreatic cancer.

## Additional files

**Additional file 1.** Identified differentially expressed genes of the merged data.

**Additional file 2.** Representative images of IHC assay. A. IHC assay of COL11A1 B. IHC assay of GJB2 C. IHC assay of CTRL.

## Abbreviations

TCGA: The Cancer Genome Atlas
DEGs: differentially expressed genes
GEO: Gene Expression Omnibus
GSEA: Gene Set Enrichment Analysis
GO: Gene Ontology
KEGG: Kyoto Encyclopedia of Genes and Genomes
PPI: protein–protein interaction
ROC: receiver operating characteristic curve
COL11A1: collagen alpha-1(XI) chain
GJB2: gap junction beta-2 protein
CTRL: chymotrypsin-like protease CTRL-1
IHC: immunohistochemical
DAVID: Database for Annotation, Visualization and Integrated Discovery
CC: cellular component
BP: biological process
MF: molecular function
ECM: extracellular matrix
EGF: epidermal growth factor
