# Supplementary figures and images for: Integrated whole genome microarray analysis and immunohistochemical assay identifies COL11A1, GJB2 and CTRL as predictive biomarkers for pancreatic cancer
Source: Cancer Cell Int. 2018 Nov 6;18:174. doi: 10.1186/s12935-018-0669-x (PMC6219000; doi:10.1186/s12935-018-0669-x)

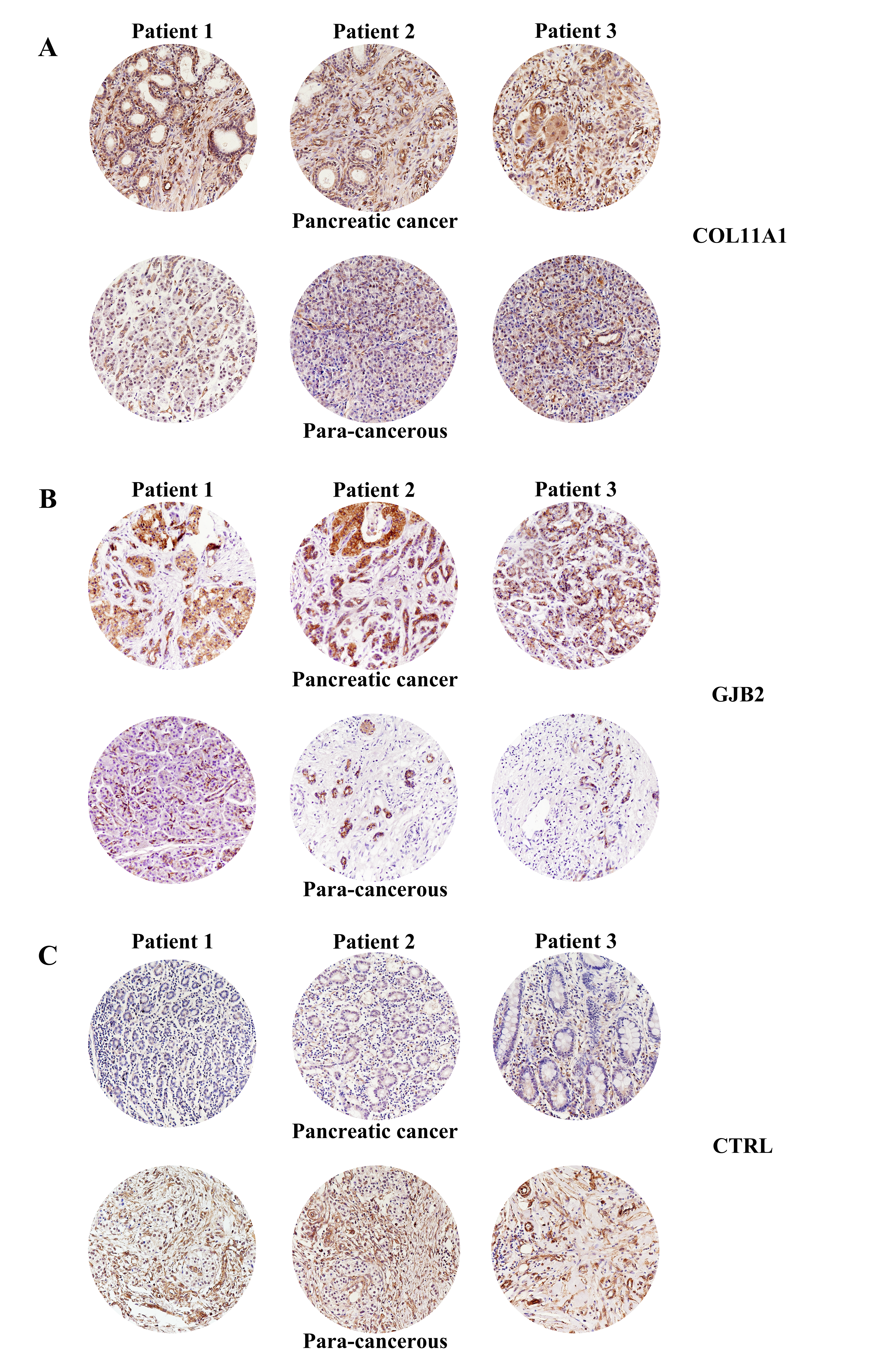

Supplement: Supplementary file 2 — Additional file 2. Representative images of IHC assay. A. IHC assay of COL11A1 B. IHC assay of GJB2 C. IHC assay of CTRL. [file 12935_2018_669_MOESM2_ESM.tif]
